# Pinned orbital moments – A new contribution to magnetic anisotropy

**DOI:** 10.1038/srep25517

**Published:** 2016-05-06

**Authors:** P. Audehm, M. Schmidt, S. Brück, T. Tietze, J. Gräfe, S. Macke, G. Schütz, E. Goering

**Affiliations:** 1 Max Planck Institute for Intelligent Systems, Heisenbergstr. 3, D-70569 Stuttgart, Germany; 2 Physikalisches Institut, Universität Würzburg, Am Hubland, D-97074 Würzburg, Germany; 3 Quantum Matter Institute and Department of Physics and Astronomy University of British Columbia 2355 East Mall, Vancouver, V6T 1Z4, Canada; 4 Max Planck Institute for Solid State Research, Heisenbergstraße 1, D-70569, Stuttgart, Germany

## Abstract

Reduced dimensionality and symmetry breaking at interfaces lead to unusual local magnetic configurations, such as glassy behavior, frustration or increased anisotropy. The interface between a ferromagnet and an antiferromagnet is such an example for enhanced symmetry breaking. Here we present detailed X-ray magnetic circular dichroism and X-ray resonant magnetic reflectometry investigations on the spectroscopic nature of uncompensated pinned magnetic moments in the antiferromagnetic layer of a typical exchange bias system. Unexpectedly, the pinned moments exhibit nearly pure orbital moment character. This strong orbital pinning mechanism has not been observed so far and is not discussed in literature regarding any theory for local magnetocrystalline anisotropy energies in magnetic systems. To verify this new phenomenon we investigated the effect at different temperatures. We provide a simple model discussing the observed pure orbital moments, based on rotatable spin magnetic moments and pinned orbital moments on the same atom. This unexpected observation leads to a concept for a new type of anisotropy energy.

The unidirectional anisotropy in ferromagnet/antiferromagnet (FM/AFM) systems is a prominent example of an interface-driven macroscopic magnetic effect. Cooled in an external magnetic field the magnetic hysteresis loop in such an exchange bias (EB) system shows a horizontal shift (HB)^1^,^2^. It was shown that in these systems, a fraction of pinned uncompensated magnetic moments is not rotating with the external field^3^,^4^,^5^. In general, magnetic moments in solids are a combination of spin and orbital contributions. The magnetic interaction with the crystal is based on orbital moments, trying to align the spin moments parallel (antiparallel) for more (less) than half band filling by Spin-Orbit-Coupling (SOC)^6^. To understand the underlying mechanism we investigated the spectroscopic nature of pinned magnetic moments using a Co/FeMn-EB system. Here, we use X-ray magnetic circular dichroism (XCMD) to separate spin and orbital magnetic moments^7^ for the pinned uncompensated magnetic moments in an element specific way.

## Experimental

**Figure 1:**
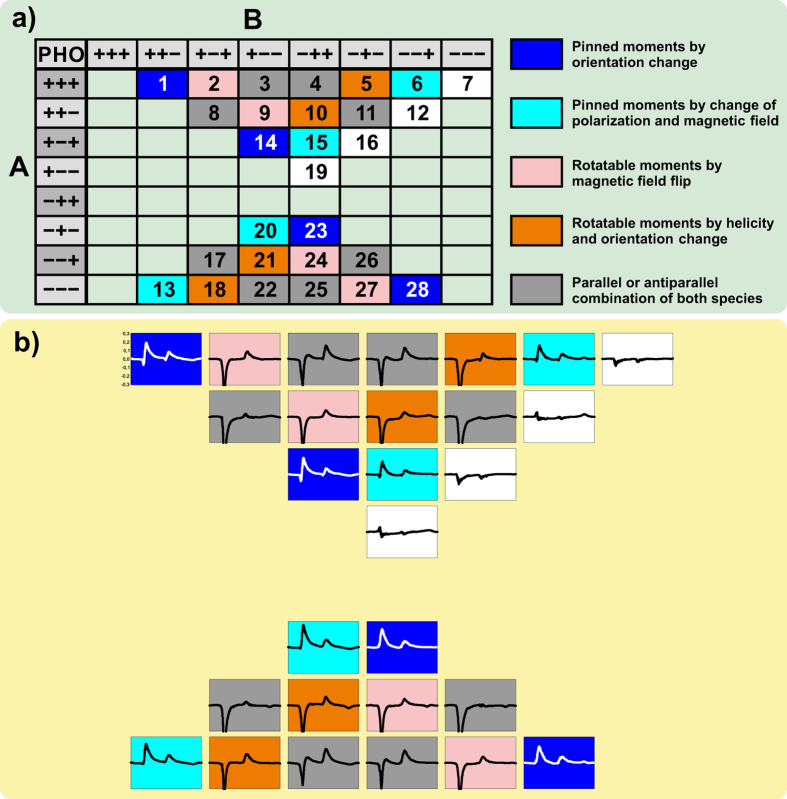
(**a**) is an overview of all possible configurations of polarization, external magnetic field, and sample orientation. The first “+” or “−” sign is for either left or right circular polarization (P) of the X-rays. The second sign is assigned to the direction of the magnetic field (H) (“+” or “−” for north or south). The last sign defines the orientation of the sample where “+” stands for θ = 41° and “−” for θ = 139°. All non-zero combinations of these eight datasets are shown. The XMCD is constructed by subtracting the right side (A) from the upper side (B). The resulting 28 different XMCD spectra (A-B) are sensitive to various types of magnetic moments. The color code is chosen in such a way that the XMCD for the same type have the same color. Pinned magnetic moments are received by just changing the orientation of the sample shown in dark blue (No. 1, 14, 23, 28), while the light blue ones are related to change in polarization and field (No. 6, 13, 15, and 20). The spectra for the rotatable moments are determined by a change of the magnetic field, shown in pink (No. 2, 9, 24, 27), and also by a change of the polarization and of the orientation, which is here color coded in orange (No. 5, 10, 18, 21). For the grey scale background it is a combination of parallel (No. 8, 11, 17, 26) and antiparallel (No. 3, 4, 22, 25) orientation of pinned and rotatable magnetic moments. Subfigure (**b**) shows icons of the corresponding measurements for 135 K in the same scheme, and the same scale.

The well investigated EB system Co/FeMn was chosen^8^ because of the strong EB effect and the possibility to measure the magnetic relevant absorption edges with soft x-ray absorption and reflection techniques. An ultra-thin Co layer on top (3 nm) has been used to provide a sizable total electron yield X-ray absorption (XAS) signal from the 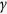-FeMn^9^ AFM below while still getting a strong EB. We used our dedicated reflectometer setup ERNSt^10^, enabling high precision sample positioning, incidence angle variation, and reproducibility. The XMCD effect is related to a change in the scalar product between the atomic magnetic moments and the axial vector of the circular light polarization, which is achieved by rotating the sample magnetization by external magnetic fields or more simply by a rotation of the sample itself. Another common way is flipping the circular light polarization. In all cases the change in the scalar product is maximized for parallel and antiparallel orientation between the light polarization and the magnetization components of interest. In the case of the EB system presented here, there are two types of uncompensated components present: rotatable and pinned magnetic moments. In the standard XMCD only the sample magnetization is flipped by an external field and is hence providing spectroscopic information on the rotatable magnetic moments only. The non-rotatable moments are not visible because they are vanishing in the difference spectrum of the two measurements. For a deeper insight in the XMCD measurements the following section is explaining various ways of deriving the difference needed to get a XMCD signal. Details are presented with respect to the expected results. In this work the XMCD measurements consists of three degrees of freedom. These are the polarization (P), the external field (H), and the sample orientation (O), as denoted by a set of three (+) or (−) signs. The XMCD signal is then resulting from the difference between two measurements (A-B). Figure 1a shows all possible configurations of XMCD results sorted by color to represent pinned (light blue, blue) or rotatable (pink, orange) moments. The order at the chart (Fig. 1a) ensures that the difference of A-B is consistent for the total orientation of the magnetic moments regarding the direction (positive or negative) of the XMCD spectrum^11^. The external field (H) defines the direction of the rotatable moments, while the sample orientation (O) is defining the direction of the pinned moments. In the majority of magnetic systems, where only rotatable uncompensated magnetic moments are present, the XMCD effect for the whole sample magnetization could also be measured by a fixed magnetic saturation of the sample in one direction and a change in circular light polarization. The system presented here is different, because the pinned moments and the rotatable moments can be aligned parallel or antiparallel with respect to each other, providing a mixed XMCD spectrum composed of both types. Because of this, the XMCD spectra in Fig. 1a are indicated in grey if the scalar product of both types is changed. Pinned and rotatable moments are parallel if “H” and “O” are in the same direction (++ or −−). The difference in parallel (Number 3, 4, 22, and 25 in Fig. 1a) or antiparallel (Number 8, 11, 17, and 26 in Fig. 1a) configuration is measured and shown in the supplemental material. In order to be sensitive to only pinned or rotatable moments, it is required to change only one scalar product.

For detecting rotatable moments the standard method by the change of the magnetic field is still valid. If the circular polarization is changed the orientation has to be changed, too.

The detection of pinned moments is achieved by two different sorts of XMCD methods. One is the sole rotation of orientation. The other is the change of both “P” and “H”^3^. For both methods only the scalar product of the pinned moments is changed while the scalar product of the rotatable moments is unchanged for both configurations and disappears when building the difference signal.

With this large set of different XMCD configurations one has the opportunity to distinguish between pinned and rotatable moments and also identify possible measurement artifacts^12^.

## Results-XMCD

**Figure 2:**
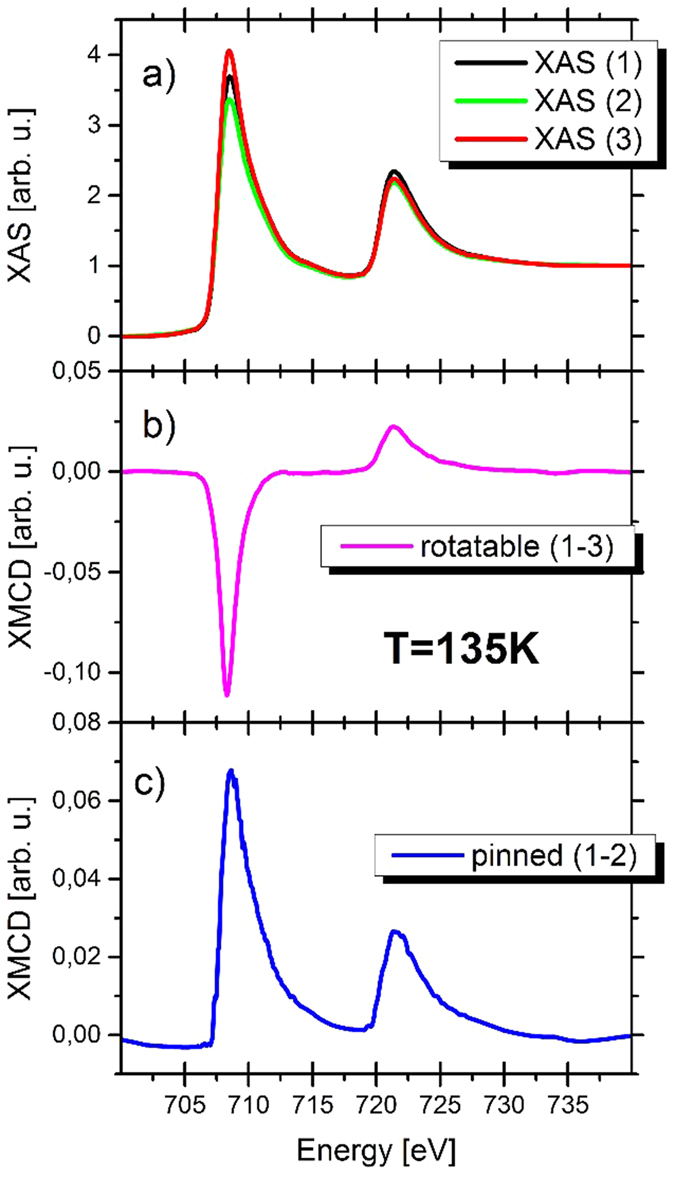
Comparison of pinned and rotatable moments at 135 K at the Fe L_2,3_ edges. The XAS signals from three different measurements are shown in (**a**), where “1” is measured with positive polarization, positive external field, and positive orientation. For “2” only the orientation is switched and for “3” only the external field is changed with respect to “1”. (**b**) Spectra of pure rotatable moments as a difference of “1” and “3”, where only the magnetic field is flipped. (**c**) Pure pinned moment spectra determined from a change in orientation (“1”-“2”).

Figure 2 depicts the energy-dependent magnetic absorption at the resonant 2p →3d transition (L_2,3_ edges) of iron with fixed circular X-ray polarization. The Fe L_2,3_ XAS and XMCD spectra have been measured at 135 K, well below the Néel temperature of the antiferromagnet. At this temperature the EB effect is strong and the signal to noise ratio is high. Figure 2a shows three different XAS spectra. With respect to the measurement “1” the sample orientation is flipped in measurement “2”, whereas in “3” the magnetic field is reversed. In order to be sensitive to the pinned moments only, spectra “1” and “2” have been subtracted. Rotatable moments are detected by subtracting spectra “1” and “3”. The field aligns the magnetic moments of the ferromagnetic Co and hence the rotatable moments in the AFM. The rotatable moment spectrum exhibits the typical shape of spin dominated metallic Fe spectra^8^,^13^, as shown in Fig. 2b. Application of the sum rules^14^ provides an orbital to spin moment ratio (m_L_/m_S_) of 0.34 for rotatable moments, which is already significantly enhanced compared to pure Fe-metal literature value of 0.043^13^. But a higher value of the ratio is expected^15^ because of the thin layer.

**Figure 3:**
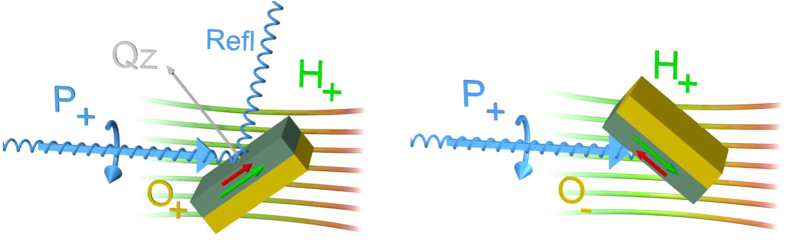
Possible configurations of the three relevant experimental vectors. The blue arrow depicts the polarization of the X-ray beam which is either positive (+) or negative (−). The external field H is defined as (+) for north or (−) for south. The samples magnetization is oriented along the field direction but confined in the sample plane, due to form-anisotropy. The sample orientation O is either defined as (+) for θ = 41° or (−) for θ = 139° with respect to the incoming beam. The presented example shows a change of the orientation from “+” to “−”. The resulting XMCD spectrum is only sensitive to pinned moments (red arrow in the sample) because the scalar product of the rotatable moments (green arrow in the sample) with the polarization vector does not change and therefore cancels out. Additionally, the reflected beam (blue) and the momentum transfer of the sample (grey), labeled as Q_z_, are depicted on the left sketch.

To identify the pinned magnetic moments, the sample orientation was flipped between two mirror orientations with fixed external field and light polarization^16^. Both experimental configurations are sketched in Fig. 3. In both cases, the rotatable moments (green arrow) are aligned at the same angle with respect to the photon beam direction, but mirrored along the plane of incidence by the sample rotation. On the contrary, the pinned moment’s projection along the photon beam direction (red arrow) is reversed in sign. Therefore, only the pinned moments are visible in the XMCD signal^3^ as shown in Fig. 2c. Surprisingly, shape and sign of this spectrum are completely different compared to conventional spectra of rotatable moments. Application of the sum rules^14^ reveals nearly pure orbital moments, reflected by the fraction of 97,5% pure m_L_ or a ratio of 39/1 (m_L_/m_S_), which is the largest value observed for transition metals so far. This value of the ratio is so high because of the nearly vanishing spin moment.

To verify this huge and quite unexpected m_L_/m_S_ ratio for the pinned moments additional measurements are presented below. First, we measured the pinned moment XMCD in all configurations showed in Fig. 1. Second, we monitored the temperature dependent correlation between the pinned moments and the EB. And third, we investigated the energy dependent Fe X-ray resonant magnetic reflectometry (XRMR) in the constant-Q_Z_ mode^17^.

First and in order to show the small amount of the residual errors in the spectra, we present in Fig. 1b sketches of all the possible XMCD spectra measured at 135 K, which could be derived from the manifold of difference spectra between the eight possible XAS spectra by switching magnetic field, light helicity and sample position. All pinned moment difference spectra show the same character and direction of the pinned moments, clearly excluding any possible artefacts. The observed pinned moment XMCD spectra are orbital moment like in shape and therefore providing double height at the L_3_ edge with respect to the L_2_ edge. This is the general shape of the XAS spectra, but without the edge jump which would be a partial hint for artefact like structures in the XMCD spectrum obtained. Indeed, this is not the case here. As this is not a hard exclusion for the presence of other tiny artefacts, we want to additionally consider the spectra where the scalar products between helicity and both magnetic moments are not changed. In this case the related XMCD spectrum should be a flat line. These are the spectra with numbers 7, 12, 16, and 19 in Fig. 1b. All those white labelled spectra are a very good measure for the amount and strength of remaining possible artefacts, and in our case, clearly far below the observed intensities determined in the pinned moment spectra.

**Figure 4:**
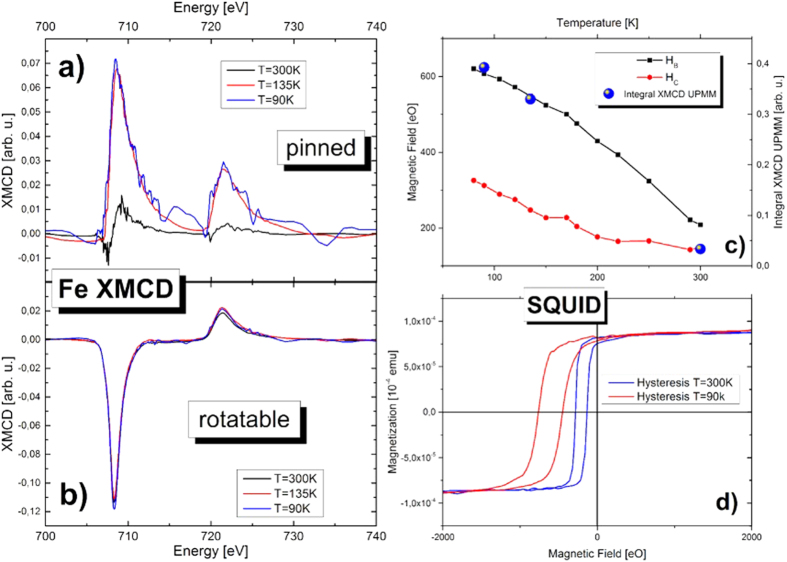
Comparison of pinned and rotatable moments at the Fe L_2,3_ edges for different temperatures. In (**a**,**b**) XMCD signals measured in absorption are plotted over the photon energy. The measurements were repeated for three different temperatures. In (**a**) the separated pinned magnetic moments are shown for different temperatures, while the rotatable magnetic moments in (**b**) are mostly temperature independent. The signal received at 300 K in (**a**) is very small and enhances for decreasing temperatures. In (**c**), the area under the pinned moment spectra is compared to temperature dependent measurements of the EB by SQUID. In (**d**) two examples of SQUID hysteresis loops are shown.

The second verification is based on the temperature dependence. Since the EB effect increases for lower temperatures and nearly vanishes at room temperature in our samples, we investigated the pinned moment’s spectra as a function of temperature. The signal, as shown in Fig. 4a, exhibits a dramatic decrease at room temperature. In contrast, Fig. 4b shows nearly temperature independent XMCD of the rotatable moments, revealing only a very small increase in the magnetic moments while cooling the sample. Figure 4c shows a comparison of the EB field HB and the coercivity HC with the strength of the pinned moment XMCD signal as a function of temperature. The values of HB and HC have been determined from SQUID measurements as shown in Fig. 4d. A clear correlation between HB and the pinned moment’s signal could be observed, confirming the reliability of the presented data.

**Figure 5:**
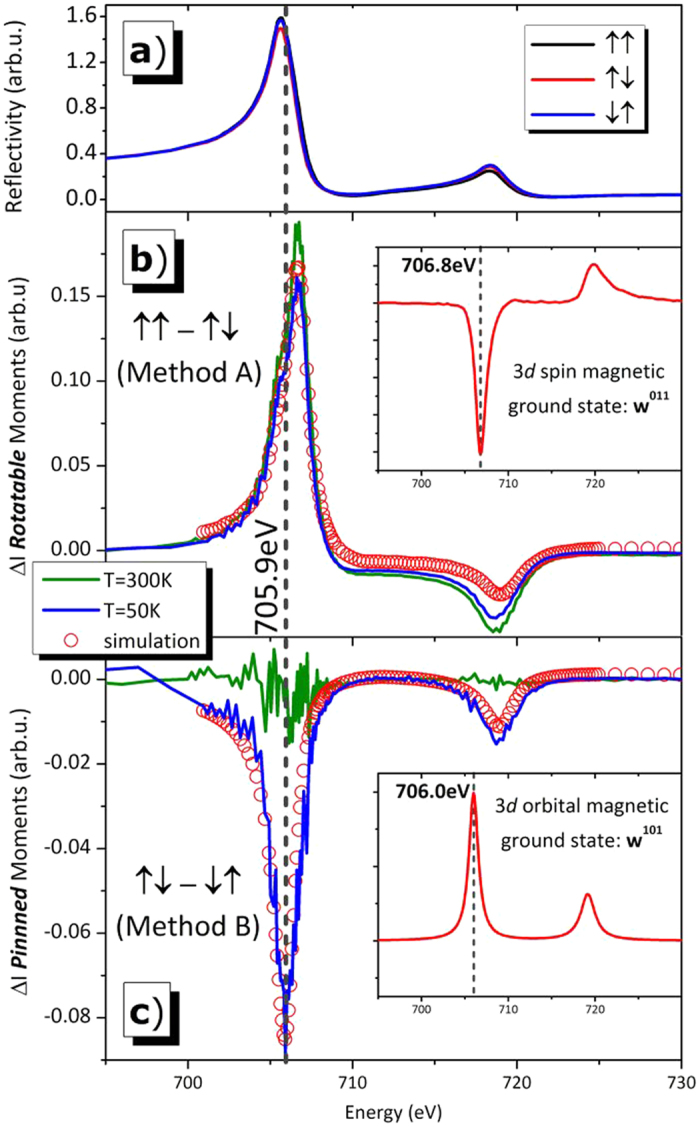
(**a**) Energy dependent circular polarized reflectivity at a constant Qz = 0.155 Å^−1^ of a 50 Å FeMn sample measured at T = 300 K. The three curves correspond to a parallel (↑↑) and an antiparallel (↑↓, ↓↑) alignment of the x-ray polarization (the first arrow) with respect to the external magnetic field (the second arrow). Subfigure (**b**) shows the difference signal related to rotatable magnetic Fe moments at T = 300 K (the green curve) and T = 50 K (the blue curve) along with a simulation (the red open circles) of the signal at T = 50 K. In subfigure (**c**), the signal from the pinned, uncompensated Fe moments is shown for both temperatures along with a simulation of the T = 50 K measurement. The indicated energy of E = 705.9 eV illustrates the different positions of the three peaks and shows that the peak maximum of the pinned moments is shifted to smaller energies compared to the rotatable ones. The insets in (**b**,**c**) show the absorption spectrum used to derive the magneto-optical constants for simulating the reflectivity spectra: for the rotatable part, a typical Fe L_2,3_, XMCD spectrum was used (related to a predominant spin moment w011), whereas the pinned spectrum simulation needs Fe 3d orbital moment (w101)-related optical properties^11^.

The third argument is based on reflection measurements. According to recent investigations, the pinned magnetic moments are located in the proximity of the AFM/FM interface^3^,^18^,^19^,^20^. In order to increase the sensitivity to the related signal, energy dependent XRMR measurements at the Fe L_2,3_ edges were performed in the constant Q_Z_-mode, as shown in Fig. 5. This pure photon-in-photon-out method provides similar spectroscopic information about the pinned and rotatable moments as the XMCD measured in absorption, but comes along with higher interface sensitivity and a better signal to noise ratio for buried systems^21^,^22^. For more details on this complex method we would like to refer to a recent review article and the examples and references provided therein^17^. Figure 5 shows magnetic reflection measurements for two different temperatures (RT and 50 K). The 50 K spectra (Fig. 5b,c) show similar results as the XAS/XMCD spectra shown in Fig. 2. Utilizing our own reflectivity matrix formalism based software package ReMagX^17^ we were able to perfectly simulate the magnetic XRMR spectra for the rotatable and pinned moments and to determine the underlying optical constants. We want to emphasize that the line shapes of the imaginary part of the simulated pinned moment optical properties are providing quite similar width and height relations with respect to the spectra derived by XMCD. Both, measurement and simulation are shown in Fig. 5b,c. The absorption based XMCD spectra and the XRMR mode determined absorptive part of the X-ray optical constants are in perfect agreement, clearly showing that the results are representing the real nature of the pinned moments. The sample used for XRMR had a thicker FM layer and a slightly thinner AFM layer, resulting in a fully vanishing EB effect at RT. Therefore, no corresponding XRMR difference signal of the pinned moments could be detected at RT, as shown by the green curve in Fig. 5c. The rotatable moment’s reflectivity signal is not decreased at RT (Fig. 5b). In summary, the analysis of the reflection data shows the consistent orbital character of the pinned moments.

## Discussion

**Figure 6:**
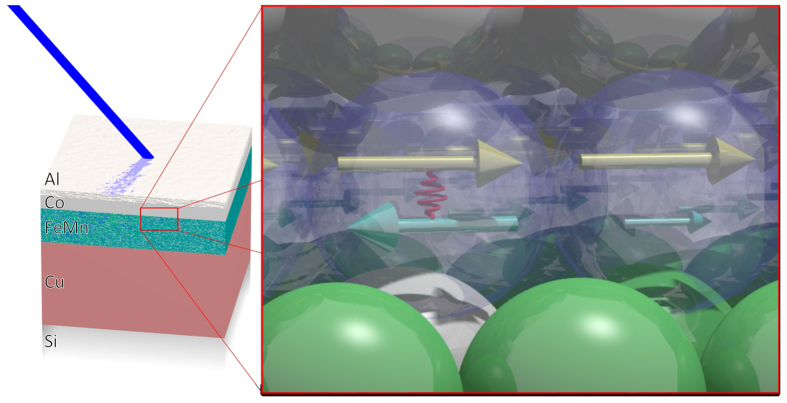
The left side shown the investigated layer setup. The TEY XMCD is sensitive especially to the Co/FeMn interface. On the right side a schematic sketch of the proposed micro model for the measured magnetic moments. Fe in dark yellow arrows representing the spin moments exchange couplet to the Co atoms above. Two different kind of orbital moments in light blue are represented, the right one is smaller because it is quenched and rotates with the spin moment. The left one is the pinned moment and is not quenched because of crystalline defect (white ball) next to it. Between the yellow spin moment and the pinned orbital moments is some energy store via the SOC. This is an intuitive model for the interpretation of the results from the various measurements in this paper.

In conclusion, we are for the first time able to show that the pinned magnetic moments in a 3d metallic EB system are of dominating orbital character. This is surprising since TM systems are usually dominated by the spin moment, related to orbital moments quenched for symmetry reasons^11^. Even in the extreme case of an atomic scenario, Fe always exhibits a larger or equal sized spin compared to the orbital moment (Hund’s rules: m_L_/m_S_ = 1 for 3d7 or m_L_/m_S_ = 0.5 for 3d6). As we have observed here, the uncompensated spins rotate with the external field, while the uncompensated orbital moments are pinned. As a working model, nearly oppositely oriented spin and orbital moments are assumed at a single Fe 3d orbital for the external field aligned against the EB direction (Fig. 6). To provide enough pinning power to overcome the Fe 3d LS-coupling, local symmetry breaking close to the AFM/FM interface is necessary. A possible source could be the presence of crystalline defects, which have been previously observed^23^ in the context of pinned magnetic moments^24^. These vacancies provide a strongly increased anisotropy energy and less quenched orbital magnetic moments. On such a side an orbital moment may be in an environment where it is not rotating with the external field. Non-collinear behavior of the expectation value of the spin and high orbital magnetic moment was predicted theoretically for systems with strong symmetry breaking^25^,^26^. Small angles between spin and orbital moments have also been observed experimentally in the context of magneto-crystalline anisotropy^27^,^28^. The rotation of the spin moment is induced by the exchange coupling to the Co layer, loading a local LS-coupling spring with a strongly pinned orbital moment, providing an additional component to the magneto-crystalline anisotropy energy. As a consequence the higher and stronger interacting orbital moment could not be rotated along the spin direction (presumably parallel or antiparallel for more or less than half filled band) for external fields applied (shown in the pinned moment spectra, which are orbital moment dominated). As the spins are strongly exchange coupled (with eV energy scale) to the FM on top, a few spins could be rotated (shown in the spin dominated spectra for rotatable moments), while the orbital moments remain pinned on these sites, because the SOC is orders of magnitude smaller compared to the exchange coupling). On positions with higher quenching and better cubic symmetry we expect a reduced orbital moment interaction with the crystal structure and the smaller orbital moment could be flipped in external field by the SOC and the rotated spins. Therefore, the rotatable moments measure here by XMCD are related to rotatable moment’s sites AND to the spin part of the pinned moment’s sites, while the pinned moments are purely related to the orbital contributions of the high orbital moment sites. As a consequence, the same 3d electrons are responsible for the spin and orbital moments, but only the orbital part is pinned.

We like to emphasize, that our observation does not offer a simple universal explanation for EB. The observed effect must not be limited to the AF-FM interface. It could be also present in many other magnetic systems. However, due to the random orientation of the pinned moments in these systems, the respective XMCD signal would be zero. This effect could also play an undiscovered but important role in disordered and spin-glass systems (see chapter 3.2 in Nogues *et al.*^2^). Owing to the induced asymmetry of the field cooling effect in EB systems, the pinned moments align in one direction and are therefore detectable by XMCD. Our discovery might open new perspectives for the general understanding of magnetic systems as well as for the tailoring of application-relevant compounds, like new TM based super magnets.

## Methods

All samples were prepared by ion-assisted RF sputtering in a UHV chamber with a base pressure of less than 5×10^−8^ mbar. The average deposition rate under working conditions was roughly 1 Å/s with an Ar partial pressure of 1×10^−4^ mbar. Si pieces with a native oxide layer of roughly 27 Å cut from a commercially bought wafer were used as substrates. A Cu buffer layer was grown on the Si to promote fcc growth of the subsequent Fe_70_Mn_30_ layer. All samples were capped by an Al protection layer to prevent oxidation. To induce a well-defined anisotropy in the sample an in-plane oriented magnetic field of 170 mT was applied during growth. The XAS-TEY measurements were performed on a sample with the following configuration: Al 2 nm/Co 3 nm/FeMn 7 nm/Cu 100 nm/Si. For the energy dependent reflectivity measurements a sample with the following layer thicknesses was used: Al 1.5 nm/Co 6 nm/FeMn 5 nm/Cu 10 nm/Si. For the temperature dependent hysteresis loops a Quantum Design MPMS-XL 7 Tesla SQUID magnetometer was used. All absorption and scattering measurements were conducted during three beamtimes at the UE56/2-PGM1 beamline at BESSY II using the custom-built UHV reflectometer experiment ERNSt^10^. The instrument allows the adjustment of the angle of incidence with a reproducibility better than 0.01° and an absolute error smaller than 0.05°. For this small absolute error in the angle we can estimate a possible rotatable moment XMCD offset signal in the pinned moment spectra, less than one order of magnitude with respect to the obtained noise level. The stability of the beam position on the sample during rotation is better than 0.1 mm. As our ERNSt system has its own beam position monitoring system, beam drift has been compensated for a few microns. The integration time per point for the energy dependent scattering spectra was 400 ms. The external field was generated by a stabilized electromagnet generating a field of 170 mT. The in plane component for the sample at 41° amounted to 128 mT.

## Additional Information

**How to cite this article**: Audehm, P. *et al.* Pinned orbital moments – A new contribution to magnetic anisotropy. *Sci. Rep.* **6**, 25517; doi: 10.1038/srep25517 (2016).

## Supplementary Material

Supplementary Information
