# Supplementary material for: Pinned orbital moments – A new contribution to magnetic anisotropy
Source: Sci Rep. 2016 May 6;6:25517. doi: 10.1038/srep25517 (PMC4858686; doi:10.1038/srep25517)
Supplement: Supplementary Information [file srep25517-s1.pdf]

Pinned orbital moments – A new contribution to magnetic anisotropy

P. Audehm[1], M. Schmidt[1], S. Brück[2], T. Tietze[1], J. Gräfe[1], S. Macke[3,4], G. Schütz[1], E. Goering[1]

[1]Max Planck Institute for Intelligent Systems, Heisenbergstr. 3, D-70569 Stuttgart, Germany
[2]Physikalisches Institut, Universität Würzburg, Am Hubland, D-97074 Würzburg, Germany
[3]Quantum Matter Institute and Department of Physics and Astronomy University of British Columbia 2355 East Mall Vancouver, Canada
[4]Max Planck Institute for Solid State Research, Heisenbergstraße 1, D-70569, Stuttgart, Germany

Supplemental information

Here we want to show and discuss briefly the measured XMCD spectra mentioned in the paper.

We are taking a close look at all the combination of subtracting two measurements and the resulting XMCD signal. Again, the $2x2x2 = 8$ measurements for switching polarization, external field and orientation where normalized (Method also showing in this supplemental) and then subtracted from each other. The result is a plot of all 28 unique XMCD signals shown in Subfigure 1:

The numbering and the coloring is consistent with Figure 1 of the Paper and also to the List of all XMCD Signals in this supplemental. The gray underlying areas (3, 4,8,11,17,26,22, and 25) can be grouped in two different ways. One is to distinguish between parallel (3, 4, 22, and 25) and antiparallel orientation (8, 11, 17, and 26) of the rotatable and pinned magnetic moments. And this is also represented by the clear difference in the shape of the measurements.

As mentioned in the paper for the conventional XMCD measurement, where the external field and therefore the rotatable moments are flipped, the scalar product for the pinned moments is not changed at all, and therefore the pinned moments are not visible in the XMCD, as indicated by the pink shaded squares (Nr. 2, 9, 24, and 27). If one wants to get exactly the same result for a flipped helicity experiment, one has to hold the external field fixed, leaving the rotatable moments fixed in space, while the pinned moments are also rotated. Therefore, the scalar product changes between the rotatable moments, where the pinned moments are again not visible, as measured in SF1 shown in the orange shaded squares (Nr. 5, 10, 18, 21). Both of the methods to measure the rotatable moments are showed in Subfigure 1. All of the XMCD spectra are showing the same behavior.

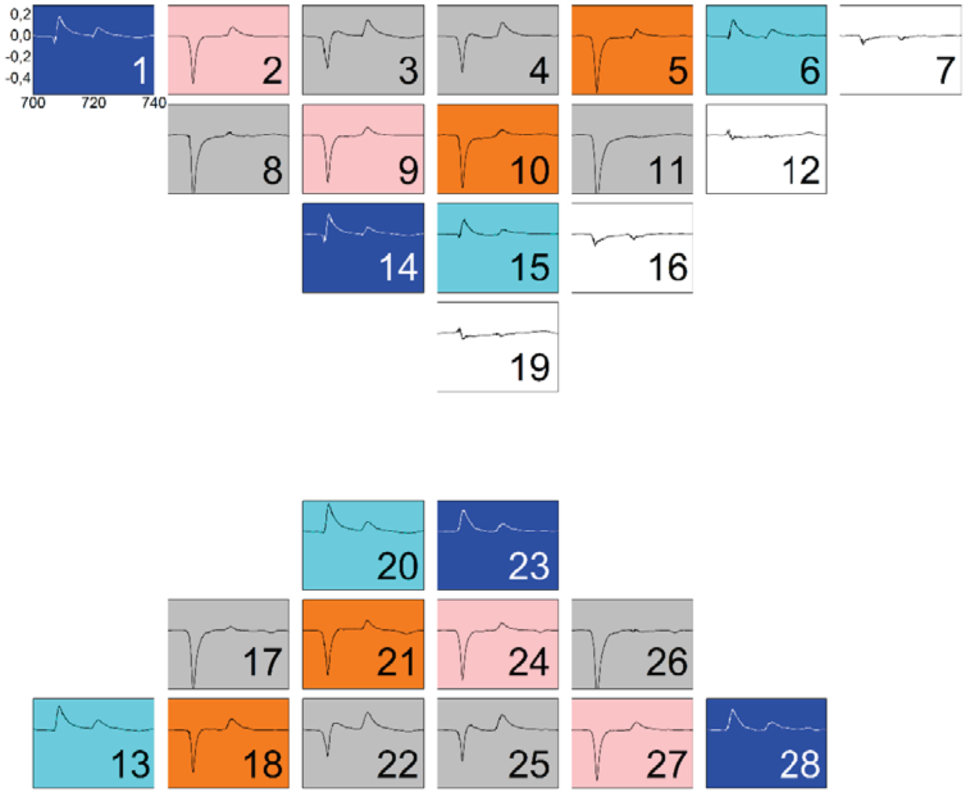

Subfigure 1 All possible XMCD measurements for 135 K. All shapes and signs at L_3 and L_2 edges are reproduced by the different XMCD spectra. Colors and Numbers are explained in the text or in the main paper.

A similar procedure could be done to detect only the pinned moments. In a fixed helicity measurement one has to fix the orientation of the rotatable moments and just change the direction of the Pinned moments. This could be done by holding the external field and the light helicity fixed and just changing the sample orientation as mentioned in SF1 by the dark blue shaded experimental options (Nr. 1, 14, 23, 28). On the other hand, an experiment which changes the light helicity has to fix the orientation of the Pinned moments, while the rotatable moments are rotated in a similar way. Therefore the scalar product of the Pinned moments changes, while the scalar product of the rotatable moments is held fixed, making them invisible for XMCD, as indicated in SF1 by the light blue shaded squares (Nr. 6, 13, 15, and 20). Also here all spectra are reproducing the expected same shape.

Following there will be a list with all the combinations of the XMCD signals possible by 8 different XAS measurements by subtracting measurement A from measurement B. The numbering is the same as in SF1.

#	A	B	fix	change	pure	combination
1	PPP	PPN	PH	O	pinned	
2	PPP	PNP	PO	H	rotatable	
3	PPP	PNN	P	HO		parallel
4	PPP	NPP	OH	P		parallel
5	PPP	NPN	H	PO	rotatable	
6	PPP	NNP	O	PH	pinned	
7	PPP	NNN	-	PHO		
8	PPN	PNP	P	HO		antiparallel
9	PPN	PNN	PO	H	rotatable	
10	PPN	NPP	H	PO	rotatable	
11	PPN	NPN	OH	P		antiparallel
12	PPN	NNP	-	PHO		
13	NNN	PPN	O	PH	pinned	
14	PNP	PNN	PH	O	pinned	
15	PNP	NPP	O	PH	pinned	
16	PNP	NPN	-	PHO		
17	NNP	PNP	OH	P		antiparallel
18	NNN	PNP	H	PO	rotatable	
19	PNN	NPP	-	PHO		
20	NPN	PNN	O	PH	pinned	
21	NNP	PNN	H	PO	rotatable	
22	NNN	PNN	OH	P		parallel
23	NPN	NPP	PH	O	pinned	
24	NNP	NPP	PO	H	rotatable	
25	NNN	NPP	P	HO		parallel
26	NNP	NPN	P	HO		antiparallel
27	NNN	NPN	PO	H	rotatable	
28	NNN	NNP	PH	O	pinned	
